# Transport of Asian surface pollutants to the global stratosphere from the Tibetan Plateau region during the Asian summer monsoon

**DOI:** 10.1093/nsr/nwaa005

**Published:** 2020-01-23

**Authors:** Jianchun Bian, Dan Li, Zhixuan Bai, Qian Li, Daren Lyu, Xiuji Zhou

**Affiliations:** 1 Key Laboratory of Middle Atmosphere and Global Environment Observation, Institute of Atmospheric Physics, Chinese Academy of Sciences, Beijing 100029, China; 2 College of Earth and Planetary Sciences, University of Chinese Academy of Sciences, Beijing 100049, China; 3 State Key Laboratory of Severe Weather, Chinese Academy of Meteorological Sciences, China Meteorological Administration, Beijing 100081, China

**Keywords:** Tibetan Plateau, atmospheric composition, troposphere–stratosphere transport, deep convection, Asian summer monsoon

## Abstract

Due to its surrounding strong and deep Asian summer monsoon (ASM) circulation and active surface pollutant emissions, surface pollutants are transported to the stratosphere from the Tibetan Plateau region, which may have critical impacts on global climate through chemical, microphysical and radiative processes. This article reviews major recent advances in research regarding troposphere–stratosphere transport from the region of the Tibetan Plateau. Since the discovery of the total ozone valley over the Tibetan Plateau in summer from satellite observations in the early 1990s, new satellite-borne instruments have become operational and have provided significant new information on atmospheric composition. In addition, *in situ* measurements and model simulations are used to investigate deep convection and the ASM anticyclone, surface sources and pathways, atmospheric chemical transformations and the impact on global climate. Also challenges are discussed for further understanding critical questions on microphysics and microchemistry in clouds during the pathway to the global stratosphere over the Tibetan Plateau.

## INTRODUCTION

Chemical species emitted at Earth's surface can be transported into the stratosphere, where they can influence climate and initiate many chemical processes responsible for stratospheric ozone variation [1–3]. Therefore, it is critical to understand how chemical species, whether natural or anthropogenic, are transported from the surface into the stratosphere.

In the global view, the stratospheric mass circulation is governed by the so-called Brewer–Dobson circulation, in which tropospheric air enters the stratosphere in the tropics, upwells within the tropical stratosphere and then spreads poleward before descending in the middle and high latitudes [4]. It is well recognized that the tropical tropopause layer (TTL) acts as a ‘gate to the stratosphere’ [5]. Although transport of air from the troposphere to the stratosphere occurs primarily in the tropics, many tropospheric pollutants cannot enter the stratosphere through this stratospheric gate [6].

Significant transport of air from the surface into the stratosphere also occurs over the Asian summer monsoon (ASM) regions. During the boreal summer, the Intertropical Convergence Zone (ITCZ) moves northward and is located between 15°N and 30°N over Asia, directly over highly populated and polluted regions of southern Asia [7]. The ITCZ, where air parcels converge at low levels, is associated with the occurrence of frequent deep convection during the ASM period. Deep convection and the associated ASM anticyclone form a critical pathway for air from the surface to enter the global stratosphere, and it is obviously a pathway strongly affected by pollutants emitted at the ground [6,8].

Once entering the upper troposphere and lower stratosphere (UTLS), pollutants accumulate and undergo chemical processes in a reactive reservoir, from which carbon-, sulfur-, nitrogen- and halogen-containing reaction products disperse globally [9]. The abundant aerosol precursor emissions from southern Asia also lead to significant aerosol formation in the UTLS within the ASM anticyclone, that is the Asian Tropopause Aerosol Layer (ATAL). Subsequently, these aerosols spread throughout the entire lower stratosphere in the Northern Hemisphere and contribute significantly to the stratospheric aerosol layer [10]. Currently, pollutant emissions are increasing rapidly in the monsoon-impacted southern region of Asia [11]. It is therefore expected that increasing emissions will intensify the pollutant flux into the global stratosphere through the ASM circulation in future decades.

In this review, we summarize the significant research findings regarding critical atmospheric processes that affect the transport of Asian surface pollutants to the global stratosphere over a broad region including the Tibetan Plateau. We discuss their potential contribution to the global budget of stratospheric chemical species and the implications for the global climate.

## FORMATION OF THE SUMMERTIME OZONE VALLEY OVER THE TIBETAN PLATEAU

Troposphere–stratosphere transport over the Tibetan Plateau was first recognized as a result of the discovery in 1994 of a ‘summertime ozone valley over the Tibetan Plateau’ by Zhou and Luo [12]. Analysis of total ozone data from the Total Ozone Mapping Spectrometer (TOMS) experiment aboard the Nimbus-7 satellite shows that total ozone has relatively low values over the Tibetan Plateau (∼10% lower than the same latitude) during May through September every year [13]. Considering the local atmospheric circulation over the summertime Tibetan Plateau, which is controlled by the South Asian High in the upper troposphere and the frequent occurrence of convection in the lower levels, Zhou *et al.* [14] speculated that the Tibetan Plateau is an important pathway for lower tropospheric air to reach the stratosphere, and that surface pollutants within the range of hundreds of kilometers around the Tibetan Plateau converge to the Tibetan Plateau, rise to the lower stratosphere and then diverge. Therefore, transport of ozone-poor air from the troposphere to the stratosphere, along with physical and chemical processes (e.g. hydrogen chloride (HCl)) in the stratosphere involving pollutants transported from the lower levels, may be the reasons for the formation of a summertime ozone valley over the Tibetan Plateau [14,15].

Since this discovery, Chinese scientists have made great efforts to characterize the features of the summertime ozone valley over the Tibetan Plateau and understand its formation mechanisms. Ozonesondes were launched in Xining (36.43°N, 101.45°E, 2296 m above sea level) during April 1995 through August 1996 [16], and in Lhasa (29.40°N, 91.03°E, 3650 m above sea level) during June–October 1998 [17,18]. These ozonesonde measurements show that the summertime ozone concentration around the tropopause over the Tibetan Plateau is lower in comparison to the ozonesonde measurements at Kagoshima Island (31.55°N, 130.55°E) of Japan at a similar latitude, in agreement with the results from satellite measurements of the Stratospheric Aerosol and Gas Experiment (SAGE) I [19], SAGE II and Halogen Occultation Experiment (HALOE) [20,21]. All of these measurements show that the ozone concentration between the 12 -and 22-km levels over the Tibetan Plateau is significantly lower in comparison to regions outside of the ASM region at the same latitude.

In the first studies, many mechanisms were suggested to explain the formation of the ozone valley. The air column above the Tibetan Plateau region is shorter because of terrain and this had been suggested as an explanation for the variation of total ozone column [22]; however, from the earliest studies, it was thought to make a secondary and minor contribution [23,24]. Other suggested mechanisms fall into two main categories: dynamical and chemical. Dynamical processes postulated to explain the ozone valley over the Tibetan Plateau included the elevated heat source associated with thermally forced circulation [23,25,26]; large-scale uplift and descent of isentropic surfaces and higher tropopause height [24], temperature and geopotential height [27]; and troposphere–stratosphere mass exchange [28–31]. Chemical processes were also investigated in earlier studies; however, they were found to play a minor role [29]. Overall, most of these mechanisms focus only on local atmospheric circulation around the Tibetan Plateau, and almost no ozone profiles were used to support the earliest analyses.

Subsequent studies have used satellite measurements of both total ozone and ozone profiles to investigate the possible mechanisms for the ozone valley formation. Based on these measurements, it has been found that the formation of the summertime ozone valley over the Tibetan Plateau is related to two factors: terrain-induced air-column variations and dynamical processes that cause UTLS ozone to be lower in the ASM region [32]. The first factor is seen by comparing the Tibetan Plateau (elevation ∼4000 m) to the Iranian Plateau (elevation ∼1000–1500 m). Both plateaus share almost the same ozone profiles in the upper troposphere and stratosphere [20], but the atmosphere above the Tibetan Plateau has ∼14 Dobson units (DU) less total ozone. The total ozone difference between the two plateaus is related to their elevation difference, with the Tibetan Plateau lacking the integrated ozone partial column from (non-mountain) ground to the mountain surfaces. Actually, along the same latitude, total ozone changes identically with variations in terrain height, showing a high correlation with terrain pressure over the whole ASM region. Calculations show that total ozone decreases by 4–4.5 DU when the pressure altitude rises each 100 hPa, and that the high elevation of the Tibetan Plateau causes a decrease of ∼20 DU total ozone in comparison to sea level [32].

Dynamical processes in the ASM region lead to a second factor that causes the ozone valley above the Tibetan Plateau. Satellite observations of ozone profiles show that ozone concentrations over the ASM region have lower values in the UTLS than those over the non-ASM region [20,32]. These observations point to dynamical processes in the ASM region as a root cause, in particular the frequent deep convective transport of low-ozone air from the lower troposphere to the UTLS region, combined with trapping by the ASM anticyclone [6]. This negative offset contributes to another ∼20 DU deficit in the ozone column over the ASM region [32].

In total, these two factors (higher terrain height of the Tibetan Plateau and lower UTLS ozone within the ASM anticyclone) would be expected to contribute to a ∼40-DU decrease in the ozone column over the Tibetan Plateau. However, measurements show that summertime total ozone over the Tibetan Plateau is only ∼33 DU lower than zonal mean values over the ocean at the same latitudes. This offset suggests that the ASM region has higher ozone concentrations in the lower troposphere (below 300 hPa), which contributes ∼7 DU of the total ozone. This explanation for the offset is confirmed by ozonesonde and satellite observations [32].

**Figure 1.**
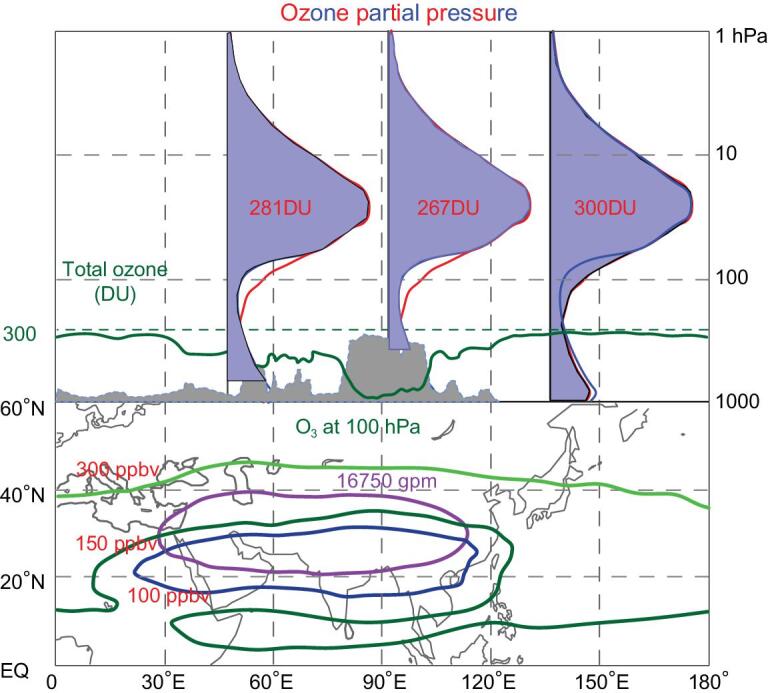
This figure summarizes the formation of the summertime total ozone valley over the Tibetan Plateau, which extends from ∼30°E to ∼120°E longitude. The upper panel shows the ozone profiles for the ASM region (blue) and the non-ASM region (red), total ozone (green) and the terrain altitude (gray shading). The lower panel shows the ozone-concentration distribution at 100 hPa (light green, green and blue for 300, 150 and 100 ppbv, respectively) and the location of the ASM anticyclone (denoted by 16 750-gpm contour, purple).

Figure 1 summarizes the formation of the summertime total ozone valley over the Tibetan Plateau. At the larger scale, the ozone profiles are different between the ASM region and non-ASM region, as seen in the plot at the far-right side of the upper panel, particularly in the UTLS. This is also evident in the ozone-concentration distribution at the level of 100 hPa (lower panel). The ASM anticyclone (denoted by the 16 750-gpm contour at 100 hPa) has lower ozone concentrations inside (blue, green and bright-green curves denote different ozone mixing ratios). The non-ASM ozone profile (red line) has a higher ozone concentration in the UTLS and lower ozone concentration in the lower troposphere, and the total column is 300 DU, ∼13 DU higher than that over the ASM region (i.e. 287 DU) when the terrain effect is removed.

For the whole ASM region, the ozone profiles are almost the same, but the Tibetan Plateau and the Iranian Plateau have 267 and 281 DU of total ozone, respectively, due to the terrain variations (the far-left and middle plots of the upper panel in Fig. 1). The Tibetan Plateau has ∼20 DU lower total ozone caused by its high elevation in comparison with the ASM region without terrain. The budget for the summertime total ozone over the Tibetan Plateau can be summarized as:

$$\begin{equation*}\mathop {267\,{\rm{DU}}}\limits_{(1)} = \mathop {300\,{\rm{DU}}}\limits_{(2)} + \mathop {7\,{\rm{DU}}}\limits_{(3)} - \mathop {20\,{\rm{DU}}}\limits_{(4)} - \mathop {20\,{\rm{DU}}}\limits_{(5)} \end{equation*}$$

where the five terms stand for the total ozone over the Tibetan Plateau (1) and the non-ASM region (2), the higher ozone column in the lower troposphere over the ASM region (3), the total ozone shortage caused by the lower UTLS ozone within the ASM anticyclone (4) and finally the terrain-induced air-column shortage (5), respectively.

## POLLUTANT TRANSPORT TO THE STRATOSPHERE VIA ASM CIRCULATION

As mentioned above, the ASM circulation is a critical factor in the formation of the summertime ozone valley over the Tibetan Plateau. However, it is also an important factor beyond this role, and at scales larger than at the Tibetan Plateau. Actually, the ASM circulation influences not only the ozone distribution, but also other aspects of atmospheric composition in the UTLS.

**Figure 2.**
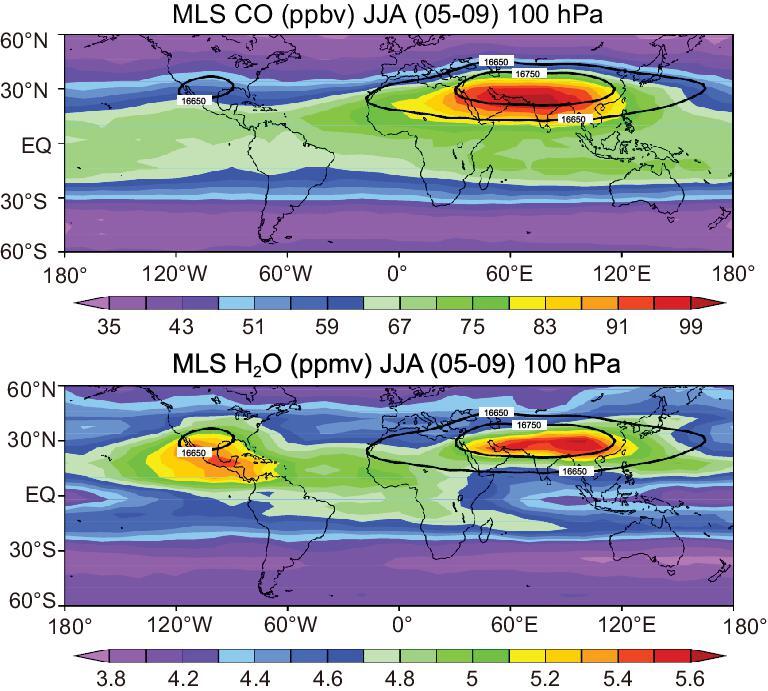
Distribution of CO (upper) and water vapor (lower) as observed by the Microwave Limb Sounder (MLS) satellite at the level of 100 hPa for the summertime average during 2005–09. The solid black curves are the contours for summertime mean geopotential height at 100 hPa. This figure is adapted from Bian *et al.* [35].

Satellite measurements show that the ASM anticyclone or the South Asian High, which covers and centers the Tibetan Plateau, creates a contained area having increased or decreased concentrations of various atmospheric constituents. The ASM anticyclone has higher concentrations of tropospheric tracers (such as carbon monoxide (CO), hydrogen cyanide (HCN) and methane (CH_4_)) [6,8,33,34] as shown in Fig. 2 for CO and water vapor at the level of 100 hPa, and lower concentrations of stratospheric tracers (such as O_3_, HCl and nitric acid (HNO_3_)) [33].

Measurements and simulations show that the water-vapor concentration in the tropical lower stratosphere is about 60% higher in the boreal summer than in the winter, and that about 75% of the total water vapor transported to the global stratosphere in the summer comes from the Asian monsoon region [36]. Additional 3D global chemical transport model simulations show that most of the pollutants entering the UTLS region, such as sulfur dioxide (SO_2_), HCN and CO, are related to the transport associated with the ASM circulation [6,37].

Taken together, these satellite data and model simulations point to the ASM circulation as an important pathway for surface pollutants to rise into the stratosphere [6]. The next section describes research regarding the possible mechanisms for this transport.

### Dynamic mechanisms for transport of pollutants to the stratosphere over the ASM region

During the boreal summer, a very strong and deep ASM circulation exists in southern Asia due to the combined effects of the continent–ocean contrast and the extreme terrain of the Tibetan Plateau. The ASM circulation includes frequent deep convection in the troposphere and a very stable and strong planetary-scale anticyclone (i.e. the South Asia High, called the ASM anticyclone thereafter) in the UTLS [38–40]. During the boreal summer, with the northward migration of the ITCZ, the ASM anticyclone is included in the northern part of the TTL [41]. Because the TTL acts as the gateway to the stratosphere for tropospheric air, various microphysical and chemical processes occurring there determine the distribution of chemical species entering the stratosphere [5].

The stratosphere above the TTL is in radiative equilibrium, i.e. positive radiation heating balances the cooling by the slow adiabatic uplift of the Brewer–Dobson circulation in the tropics. The troposphere below the TTL is featured by strong convection and negative radiative heating, and therefore has large-scale weak subsidence and sporadic strong convective updraft. Within the TTL, the vertical motion is characterized by large-scale weak ascending motion accompanied by occasional convective intrusions (∼360 K). Therefore, the atmospheric boundary layer (ABL) air can rise into the stratosphere only after entering the TTL by way of deep convective transport [42].

### Deep convective transport

As stated above, deep convective transport is a prerequisite for the ABL air to enter the stratosphere, and is also the most efficient transport mechanism. Deep convection can transport the air from the ABL quickly to the main deep outflow level (∼360 K) within tens of minutes, so this is a crucial mechanism especially for very short-lived chemical species (e.g. bromocarbons, HCl) to enter the TTL [15,43,44]. The short-lived species are important in impacting the local ozone budget near the tropopause within the ASM region [15].

From the perspective of chemical-species transport, it is very important to understand the distribution of convective clouds activity within the ASM region, particularly the main level of deep convective outflow. In the upper troposphere, even a difference of only 1–3 km in the height of the main outflow level will have a great impact. Above the zero heating rate, the large-scale upward velocity is usually very small (only mm s^−1^) so it will take a few weeks to climb a height of 1–3 km [45]; deep convective transport, however, takes only several to tens of minutes. At present, there is still great uncertainty in the quantification of the detrainment processes at the convective outflow level over the Asian monsoon region [46,47].

There is also great uncertainty in the geographical distribution of deep convective clouds activity over the ASM region, though persistent deep convection is known to be located over southern Asia and southeastern Asia, which is far from the center of the ASM anticyclone where tracers show extreme values [33]. For example, by analysis of the Tropical Rainfall Measuring Mission (TRMM) satellite data, Fu *et al.* [48] showed that the frequency of deep convection (especially for convective top heights greater than 14 km) is higher over the Tibetan Plateau and its southern slope than over the South Asian monsoon region. However, other studies (using satellite-borne cloud radar observations) suggest that the strongest convection occurs over the South Asian monsoon region, rather than over the Tibetan Plateau and its southern slope [33,49–51].

Overshooting convection events (deep convection penetrating the tropopause level), with strong upward air flow, are capable of transporting air from the ABL to the stratosphere and above the neutral buoyancy level [47,52]. Irreversible exchange across the tropopause caused by gravity wave breaking and/or turbulent mixing atop these storms can have significant impacts on chemistry and climate [53]. Convective overshooting is also an efficient mechanism to transport chemical species up to the tropopause within the ASM anticyclone [54]. However, the frequency at which overshooting convection reaches the tropopause appears to be very low [47,52] and a direct way to quantify the effect of overshooting convection on transport over the ASM region is challenging.

The future goals are to know some important properties of deep convection over the ASM region, including its geographical distribution, the frequency of overshooting convection, diurnal variation and distribution of the deep convective outflow level [50,52,55]. To achieve these goals, it may be necessary to have multi-source cloud observations, including geostationary satellites, and even to develop new satellite cloud instruments (the Global Precipitation Measurement Ku band radar), as well as to develop appropriate analytical methods to estimate the vertical transport of deep-convection events.

**Figure 3.**
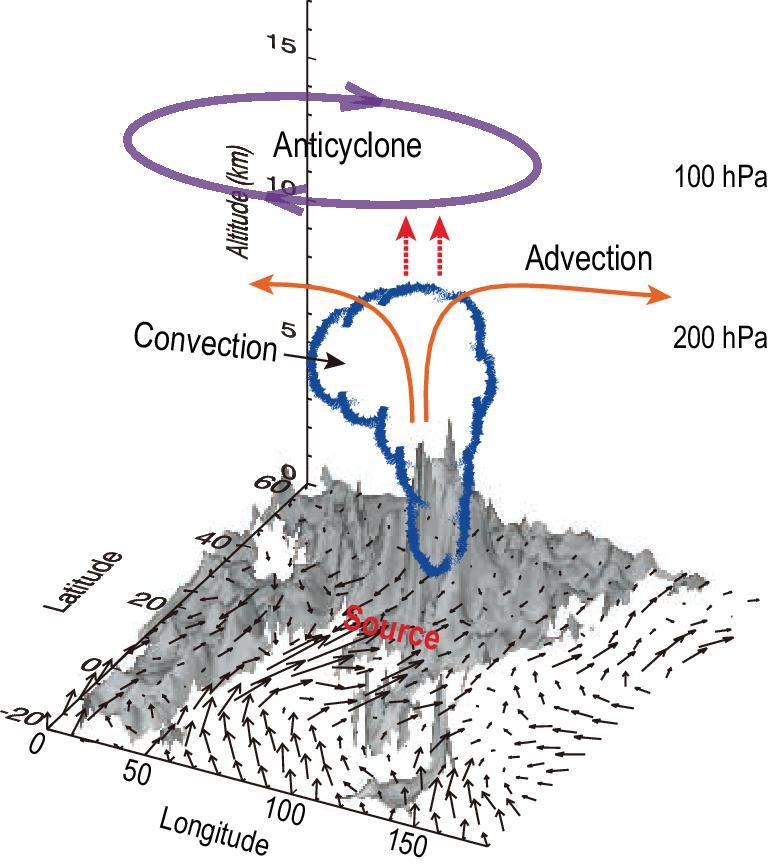
Schematic showing transport pathways to the ASM anticyclone. South Asia and Southeast Asia are denoted as the surface source region. A cloud represents monsoon deep convection. Horizontal advection at 200 hPa and the large-scale vertical transport are shown as solid and dashed red arrows, respectively. The ASM anticyclonic circulation is shown as a clockwise ellipse at 100 hPa and surface wind vectors are overlaid on top of the topography. This figure is adapted from Park *et al.* [37].

Despite the gaps in current understanding, it is known that deep convection has a great impact on the variation of atmospheric composition in the UTLS over the ASM region (as shown in Fig. 3). Both seasonal and synoptic timescale variations in water vapor and ozone within the ASM anticyclone are linked to the variations in convection intensity [42,56]. Owing to the upward transport of moist and ozone-poor air by deep convection, water vapor exhibits enhanced values and ozone shows relatively low values. If latent heating is turned off in the Weather Research and Forecasting (WRF) model to simulate the absence of deep convective transport, the observed enhanced pollutants within the ASM anticyclone are no longer reproduced [57]. Deep convection, however, can sometimes cause enhanced ozone in the upper troposphere over the Tibetan Plateau by bringing in many ozone precursors from the continental ABL of South Asia (as shown in Fig. 3), as has been observed by ozone soundings and simulated by the Chemical Lagrangian Model of the Stratosphere (CLaMS) [58–60]. The deep convective clouds over the monsoon region provide an efficient mechanism to remove air pollution via oxidation and deposition [9,61]. Organic carbon formed from reactions with volatile organic compounds during upward transport, while the number concentration decreases owing to coagulation [9]. When considering aerosol activation and removal processes above the cloud base, monsoon rains can remove aerosols efficiently [61]. Nitrogen oxides (NO_x_, or NO + NO_2_) produced by lighting associated with deep convection also contribute to chemical reactions in the UTLS [62].

The large-scale circulation of the ASM anticyclone sometimes traps the air mass uplifted by deep convection from western Pacific oceanic convection [63]. Low ozone values are measured in the upper troposphere in Lhasa and Kunming, and back-trajectory simulations show that this phenomenon is sometimes caused by rapid vertical transport associated with typhoon convections over the western Pacific Ocean and then horizontal movement to the ASM anticyclone [64–66].

### The ASM anticyclone

**Figure 4.**
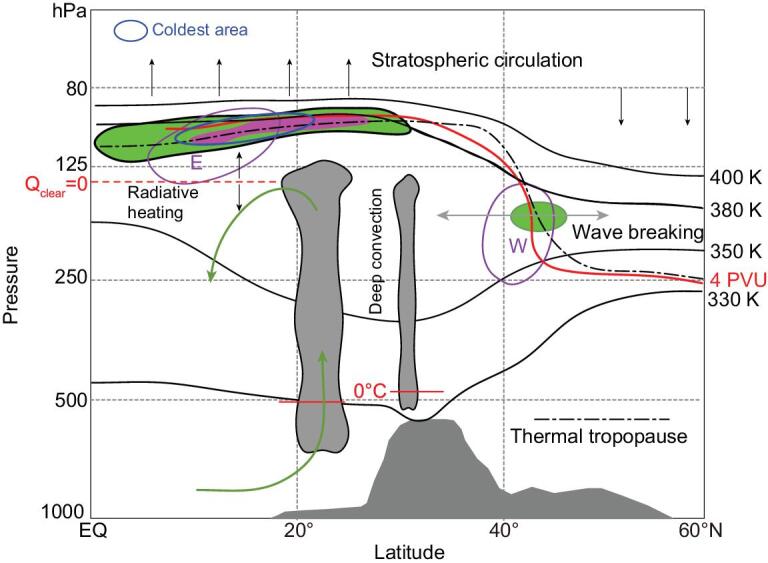
Schematic showing the pathways for tropospheric air within the ASM anticyclone to enter the stratosphere. The density of tropopause crossing points is denoted as green and pink shades at the mean temperature lapse rate tropopause (black dot-dashed line), with the pink shaded area having higher density. The coldest region (blue circle) in the UTLS is overlaid on the color-shaded area. The vertical motion is denoted as black solid vertical arrows in the UTLS. The neutral radiative heating level on the tropical side is denoted as a red dashed line. The isentropic surfaces from 330 to 400 K are denoted as black solid lines. The 4-PVU (1 PVU = 1.0 × 10***^–^***^6^ m^2^ s***^–^***^1^ K kg***^–^***^1^) is denoted as a solid red line. The stratosphere–troposphere exchange due to eddy shedding to the north of the anticyclone is denoted by the gray double arrow along the isentropic surface. The deep monsoon convections are denoted as two gray cloud towers, one for bulky convection over South Asia and another for thinner convection over the Tibetan Plateau; the freezing levels are denoted as solid red lines. The vertical monsoon circulation is denoted by the green arrows. The westerly jet and the easterly jet in the upper troposphere are denoted as pink circles. This figure is modified from Fan *et al.* [83].

Apart from the mesoscale and microscale deep convective transport, another important transport mechanism in the TTL is a slow large-scale movement combined with a horizontal mixing process. Over the ASM region, the most prominent large-scale circulation within the TTL is the gigantic ASM anticyclone (as shown in Fig. 3). The ASM anticyclone has three features that make it an important transport pathway across the TTL [42]. First, the ASM anticyclone is close to the tropical deep convections and becomes a rapid vertical-transport conduit for the air from the ABL to the TTL. Second, the closed streamlines around the ASM anticyclone have a trapping effect, which causes the surface pollutants entering the anticyclone through deep convection to stay in the interior for a relatively long time, and to be isolated and unable to exchange with the air outside the anticyclone [67]. Third, the ASM-anticyclone circulation has a relatively strong meridional wind component, which helps to promote air exchange between the tropical upper troposphere and the mid-latitude lower stratosphere [68–73]. The trapping effect of the ASM anticyclone also keeps its interior air far away from the extremely cold zone of the TTL (Fig. 4), which is located in the southeastern region of the ASM anticyclone. This has the effect of reducing the dehydration of the interior air [74] and prolonging its intense radiative heating, mainly from the radiative heating of clouds (and the ASM region has higher cirrus cloud cover in the UTLS) [42,75]. This in turn enhances vertical transport across the TTL (Fig. 4) [76].

The trapping effect of the ASM anticyclone is considered to be responsible for the UTLS composition anomalies in many atmospheric species [8,33,45,56,77]. This trapping effect also works during the intra-seasonal east–west oscillation processes of the ASM anticyclone, which causes the center of the composition extreme to move accordingly from east to west [78,79], and induces two modes in the composition distribution [80] corresponding to the two major modes of the South Asian High center (i.e. the Iranian mode and the Tibetan mode) [81]. During the Iranian mode, tropospheric tracers (such as H_2_O, CO) have positive anomalies in the UTLS over the Iranian Plateau and negative anomalies over the Tibetan Plateau, whereas stratospheric tracers (such as O_3_) show negative and positive anomalies over the Iranian Plateau and the Tibetan Plateau, respectively. The opposite situation occurs during the Tibetan mode [80]. However, there is a controversy about the two modes of the ASM anticyclone, when using the different reanalysis data and methods. Nützel *et al.* [82] showed that the bimodality of the ASM anticyclone is only found in NCEP-1 (the National Centers of Environmental Prediction) and NCEP-2 compared to other reanalysis data (e.g. ERA-Interim and JRA-55).

### Pathways from the ASM anticyclone to the stratosphere

The air entering the ASM anticyclone through deep convective transport has two fates: some will mix horizontally within the ASM anticyclone and will stay for a long time because of the anticyclone's trapping effect, and some will leave the anticyclone and enter the stratosphere.

There are two main pathways for air from the ASM anticyclone to enter the stratosphere during the boreal summertime. One pathway is that the air rises across the isentropic surface and into the tropical stratosphere through a slow diabatic uplift motion, which balances the radiative heating. This is the most important pathway, occurring mainly over the southeastern part of the ASM anticyclone (Fig. 4) [45,78,83,84]. Forward trajectory calculations show about two-thirds of the air crossing the tropopause via this pathway [83]. The greatest uncertainty in this pathway comes from the calculation of the diabatic ascent rate (see the vertical arrows in Fig. 4), because information is lacking on some key atmospheric features, especially cloud distribution [63,85].

Another pathway is that the air enters the mid-latitude stratosphere through the eddy shedding along the isentropic surface (Fig. 4) [78,83]. The subtropical westerly jet to the north of the ASM anticyclone (Fig. 4) is generally considered to be a barrier for air exchange between the anticyclone interior and the mid-latitude stratosphere. However, wave shedding caused by the anticyclone circulation and Rossby-wave interaction can take the interior air away from the anticyclone [66,86]. Even though this pathway makes a smaller contribution to air-mass transport, it is very important for causing composition changes in the stratosphere, because the horizontal gradient of composition is much larger here.

In addition, a strong equatorward airflow and a strong poleward airflow occur to the east and to the west of the ASM anticyclone, respectively. These meridional circulations lead to an in-mixing process toward the equator [70] and out-mixing process from the equator [87–91], which contributes greatly to the atmospheric composition exchange between the extra-tropical lower stratosphere and the tropical upper troposphere.

In particular, the in-mixing process associated with the ASM anticyclone can transport volcanic plumes from extra-tropical volcanic deep eruptions into the tropical stratosphere layer [92], which had been considered to be unable to contribute to the tropical stratospheric aerosol layer. This equatorward transport, through isentropic transport above the westerly jet, is primarily driven by eddy shedding. The volcanic plumes are entrained along the airflow on the eastern side of the anticyclone, are transported southwestward into the deep tropics downstream of the anticyclone and reach the TTL [92].

However, very little research has been done so far on the stratosphere–troposphere exchange caused by the interaction between the intra-seasonal oscillation of the ASM anticyclone and the outer circulation. A new project of the Asian Summer Monsoon Chemical and Climate Impact Project, supported by the National Science Foundation of the USA, is planned to take place in July–August of 2020 with the aircraft operation based in Japan. This project will address the transport pathways (vertical range, intensity and timescale) of the ASM uplifted air from inside the anticyclone to the global upper troposphere and lower stratosphere, based on the hypothesis: the western Pacific region is a major path of ASM UTLS outflow (https://www2.acom.ucar.edu/acclip).

### Simulation methods for troposphere–stratosphere transport

Two major Lagrangian methods are used to investigate the transport process from the ABL to the UTLS over the ASM region. One method is based on the distribution of deep convective cloud tops, tracing the air from the deep convective outflow levels to the TTL by running a 3D trajectory model driven by wind fields from analysis or reanalysis data sets [73,93–95]. This method does not consider the detailed transport processes and specific path within the convective clouds; rather, the air atop the deep convective outflow level is assumed to be transported directly from the ABL by deep convection. Therefore, uncertainties in this approach are mainly from cloud data and threshold parameters used to determine the tops of convective clouds [93].

Another method does not consider explicitly the distribution of convective cloud tops, but directly calculates the trajectories of the air from the ABL to the UTLS by running the trajectory model [42,63,73,96–98]. These studies focus on the geographical distribution of the ABL sources and transport pathways of the UTLS air. Obviously, this method cannot describe well the convective transport process because of relatively poor horizontal resolution. The uncertainty mainly comes from the differences among the various (re-)analysis data sets [63].

### Comparison between the Asian monsoon and the North America monsoon

The Asian monsoon and the North American monsoon are the two strongest monsoons during the boreal summer, but transport processes into the stratosphere via these two summer monsoon circulations are different. In the global water-vapor distribution (Fig. 2b) observed by the Microwave Limb Sounder (MLS) experiments onboard Aura at the level of 100 hPa during the summer (June–August) of 2005–09, there are two centers with higher concentrations of water vapor, i.e. the Asian monsoon region and the North American monsoon region, with almost the same values (5.6 and 5.4 ppmv, respectively). However, this is not the case for other atmospheric constituents, such as CO. An enhanced CO center in the globe is seen over the Asian monsoon area (Fig. 2a), but not the North American monsoon area. The observed higher concentrations of water vapor and CO over the ASM region are consistent with the above transport analysis. However, over North America, the presence of enhanced water vapor but absence of enhanced CO is still unsolved.

The enhanced water-vapor centers over both the North American monsoon region and the Asian monsoon region are considered to be caused by the upward transport of water vapor from lower levels by deep convection [36,54]. For other atmospheric constituents, however, the stronger ASM-anticyclone circulation due to stronger and deeper Asian monsoon circulation is thought to inhibit the mixing of air inside and outside the anticyclone [36], which results in a higher concentration of CO within the ASM anticyclone. In contrast, due to the weaker monsoon circulation over the North American monsoon region, the upward-lifted air by deep convection is more frequently mixed by stratospheric intrusion air from outside. Therefore, there is no enhanced CO center over North America [36]. The weaker ground source possibly contributes to the low CO. Another opinion argues that the effect of deep convection on atmospheric composition is determined by the so-called ‘convective contrast’ of specific constituents, i.e. the concentration difference of atmospheric species between the convective outflow region and the surrounding environment [54]. Because water vapor in the UTLS has larger ‘convective contrast’, the convective effect is relatively significant, and the concentration of water vapor mainly depends on deep convective transport. CO and other constituents, however, have smaller ‘convective contrast’, so the effect of convective transport on them is relatively weaker, and their concentration mainly depends on horizontal advection and Brewer–Dobson circulation transport.

The vertical difference in the water-vapor distribution maybe provides some clues for further understanding. MLS measurements show three enhanced water-vapor regions at the level of 215 hPa, namely the African monsoon region, the North American monsoon region and the Asian monsoon region, among which the water vapor over the Asian monsoon region is significantly higher than that over the other regions and the center is located over the Bay of Bengal, corresponding to the deep-convection center over the ASM region. The variation of water-vapor concentration at this level has a good correlation with the variation of convective intensity over the Asian monsoon region [33].

At higher levels, such as the 100-hPa level, the overall distributions of water vapor are similar to lower levels, but there are at least two obvious and important changes (Fig. 2b). First, the Asian monsoon region still has the highest water-vapor concentration, but the difference is not as significant. Second, the centers of the enhanced water-vapor concentration in both the Asian monsoon region and the North American region move northward. The enhanced water-vapor concentration area at the level of 100 hPa deviates far away from the deep convective region and is more easily affected by large-scale circulation. Correspondingly, the correlation between the variation of water-vapor concentration at 100 hPa and convective intensity within the Asian monsoon region is significantly decreased [33]. However, the correlation between the CO concentration at 100 hPa and convective intensity is still very high [33]. This analysis suggests that the direct transport of water vapor by deep convection to the upper troposphere decreases with the higher altitude due to the quick weakening of deep convection, and that the large-scale circulation (particularly the coldest temperature distribution) and its related dehydration process take the dominant role.

The distribution of water-vapor isotopes in the UTLS provides more information about deep convective occurrences. Water vapor containing a hydrogen isotope (HDO) observed by the Atmospheric Chemistry Experiment-Fourier Transform Spectrometer (ACE-FTS) on SCISAT shows that the North American summer monsoon region has an enhanced HDO concentration in the UTLS, but the Asian monsoon region does not [99]. The enhanced HDO concentration in the UTLS indicates the fast vertical transport of water vapor by deep convection because, during the freezing dehydration process by slow large-scale uplift, the HDO component in water vapor will first condense into liquid water droplets or ice particles and then fall out, thus the HDO component reaching the UTLS will be significantly reduced. However, the strong updraft of deep convection can transport ice particles (in which HDO content is enhanced) to the UTLS, and then some fine ice particles will sublimate and release HDO vapor in the UTLS. From the above argument, it seems that the deep-convection events in the Asian monsoon region transport less HDO vapor to the UTLS than the events in the North American monsoon region. However, it should be noted that the Asian monsoon region has much lower temperatures in the UTLS region than the North American monsoon region, and consequently significantly higher coverage of cirrus clouds [100,101]. Therefore, one possibility is that ice particles in the cirrus clouds over the Asian monsoon region contain more HDO (as H_2_O favors the gaseous state while HDO favors the condensed state) but, due to the lower temperature, these solid HDO particles do not sublimate to vapor and hence are not detected by satellite remote sensing [35].

In order to investigate the above possibility, the distribution of total water content (water vapor + ice water content) and the contribution of ice water to the total water content are analysed [102]. MLS measurements show that the distribution of total water content is different from the distribution of water vapor alone. At the level of 215 hPa, the total water content and water vapor have similar enhanced areas, and the contribution of ice-water content to the total water content is <15%. At higher levels, however, the enhanced center for total water content is closer to the deep-convection area than that for water vapor, particularly over the Asian monsoon region, and the contribution of ice-water content to the total water content is about 50% [102], which suggests the importance of ice-water content.

Next, the efficiency of transport into the stratosphere via the Asian monsoon circulation and via the North American monsoon circulation is compared. Based on the CLaMS model driven by reanalysis data, artificial tracers are released at different levels from the middle troposphere to the lower stratosphere in both monsoon regions during the summer and are tracked until the following summer [103]. Simulations show that the air-mass contributions from the ASM to the tropical pipe are about three times larger than the corresponding contribution from the North American monsoon, and the transport efficiency of the ASM is almost twice that of the North American monsoon [103].

## CHEMISTRY WITHIN THE ASM ANTICYCLONE

Previous studies of troposphere–stratosphere exchange over the ASM region have focused mainly on dynamical transport processes, while little attention given to the chemistry that occurs during the transport process. In the earliest studies, just after the summertime ozone valley over the Tibetan Plateau was discovered in 1994, Zhou *et al.* [14] had speculated in a 1995 study that the chemical reactions of pollutants transported from lower levels might have some impact on the ozone concentration in the stratosphere. However, in 2003, the model simulations of Liu *et al.* [29] found that the chemical processes related to stratosphere–troposphere exchange over the ASM region have no significant impact on stratospheric ozone. For the next few years, chemical processes were reported in the related studies [15,104]. The heterogeneous chlorine processing near the monsoon region and the reduction of NO_x_ Asian emissions have the potential to decrease ozone abundances near the tropopause.

### Discovery and formation of the ATAL

It was not until 2011 that chemical processes gained attention again in the UTLS over the ASM region. Using observations from the satellite-borne Cloud-Aerosol Lidar with Orthogonal Polarization (CALIOP), Vernier *et al.* [105] found that there exists an enhanced aerosol layer near the tropopause over the Asian monsoon region every summer, which is called the ATAL. The ATAL ranges from the eastern Mediterranean Sea (extending southward to northern Africa) to the western part of China (southward to Thailand), and its vertical extent ranges from 13 to 18 km (360–420 K of the isentropic surface), corresponding to the ASM anticyclone. Due to the low signal-to-noise ratio of CALIOP, the Compact Optical Backscatter Aerosol Detector sondes were launched over the Tibetan Plateau to confirm the findings from CALIOP and SAGE II [106]. Balloon-borne sensor measurements also show that a robust enhancement in aerosol concentration extends up to 2 km above the tropopause [10,107]. This aerosol layer is different from the stratospheric aerosol layer (i.e. the Junge layer), which is mainly affected by natural processes such as volcanic eruptions (especially in the tropics), while the ATAL is mainly formed from anthropogenic pollutant emissions rather than volcanic eruptions [105] or carbonyl sulfide [108]. However, the ATAL is sometimes concealed in the plumes from volcanic eruptions, such as the Sarychev volcanic eruption in the Kuril Island chain in June 2009 [92] and the Nabro volcanic eruption in the northeast African nation of Eritrea in June 2011 [109,110]. After these two volcanic eruptions, the erupted plumes flowed around the ASM anticyclone and made the ATAL inconspicuous.

Some model simulations show that the existence of the ATAL seems to be closely related to the upward transport of surface pollutants in the Asian monsoon region and subsequent microphysical and microchemical processes [111–113]. Due to the lack of *in situ* observations, the chemical composition of aerosols in the ATAL is not clear. Therefore, these simulation studies have made different assumptions about the composition of the ATAL aerosol. Neely *et al.* [112] assume that the ATAL is mainly from sulfate aerosols formed by the oxidation of SO_2_ (similar to the formation of the Junge layer), after which Yu *et al.* [113] add some primary and secondary organic aerosols. Mineral dust is also believed to be an important composition of the aerosol particles [114–117]. Taking multiple surface emissions into account, Gu *et al.* [111] show surprisingly that nitrate contributes up to 60% to the ATAL.

More unexpectedly, the latest satellite observations show significant amounts of gas-phase ammonia (above 15 pptv) over the subtropical regions of the southeastern Asian continent (20–30°N, 70–110°E) at the altitude of the ATAL [118,119]. Ammonia (NH_3_), primarily emitted from land surfaces, has a lifetime of only several hours due to its high affinity for water, which facilitates its effective removal via atmospheric scavenging and its incorporation in aqueous and acid particles. Gas-phase concentrations of ammonia are expected to be extremely low in the UTLS according to the wet settlement [120]. A cross-scale modeling study that includes molecular dynamic simulations and a global chemistry transport model shows that the unexpectedly high concentrations of NH_3_ in the UTLS are from the NH_3_ dissolved in liquid cloud droplets, which is released into the UTLS upon freezing and subsequent collision of ice particles during deep convection [120]. In the UTLS, the presence of NH_3_ strongly favors new particle formation through the stabilization of sulfuric-acid clusters at low temperatures due to its alkalinity. A more recent study using satellite observations and high-altitude aircraft measurements shows that solid ammonium-nitrate particles are ubiquitous in the ATAL, and that the presence of ammonium-sulfate impurities allows the crystallization of ammonium nitrate even under conditions of high relative humidity, which prevail within the ASM anticyclone [121].

Preliminary measurements have characterized the aerosol-size distribution and volatility of the ATAL. Balloon-borne *in situ* measurements show a relatively high concentration of aerosol (up to ∼50 or 25 cm^−3^) near the tropopause with diameters larger than 0.14 or 0.18 μm, and the aerosol size in the ATAL is generally <0.3 μm [10,107]. The aerosol volatility measurements (heated to a temperature >180°C) show that 80%–95% of these aerosols have a volatile component, and these aerosols are thus expected to be partially or totally liquid [107]. However, satellite remote sensing and aircraft *in situ* measurements find crystalline ammonium nitrate in the ATAL [121]. The volatility of ammonium nitrate is a possible explanation for the detection of the 80%–95% volatility fraction.

Long-term observations from satellite-borne instruments show a significant growth trend for the ATAL [106]. Ten years of SAGE II solar occultation data (1996–2005) and 8 years of Cloud-Aerosol Lidar and Infrared Pathfinder Satellite Observation (CALIPSO) lidar observations (2006–13) (after correction for the attenuation effects by Rayleigh scattering and ozone absorption, removal of cloudy pixels in the upper troposphere using a volume depolarization ratio threshold of 5% and exclusion of the periods affected by volcanic eruptions) show that there is no evidence of the ATAL in SAGE II data before 1998 but that the ATAL obviously exists after 1998 [122], and that the aerosol optical depth of the ATAL increases from ∼0.002 to ∼0.006 from 1996 to 2013 [106]. Whether the ATAL exists or not before 1998 is still a disputed question. It should be noted that the effect of the 1991 eruption of the Mt. Pinatubo volcano on stratospheric aerosols persisted until 1998 [123], which may influence the ATAL detection before 1998. The existence of the ATAL in 1997 is already there based on satellite observation [112].

### Climate impacts of the ATAL

The ATAL has many impacts on regional and even global climate. First, like aerosol particles at lower levels, the tropopause aerosols change the radiative energy budget by scattering solar radiation. Radiative calculations show that the ATAL exerted a short-term regional forcing of −0.1 W m^−2^ at the top of the atmosphere during 1996–2013 [106].

Second, particulate ammonium nitrate and ammonium sulfate can potentially promote heterogeneous ice formation by acting as ice-nucleating particles, thereby affecting the radiative properties of cirrus clouds in the upper troposphere [124]. Satellite data analysis shows that enhanced pollutant emission over Asia can increase water-vapor flux into the stratosphere in summer [125]. Satellite measurements show that, in comparison to clean areas, polluted areas have ice particles of smaller effective radius in the TTL, and higher temperature and more water vapor in the cirrus clouds. Accordingly, it is speculated that increased aerosols (as ice nuclei) will increase the number concentration of ice particles in cirrus clouds, which will reduce the radius of particles and prolong their stay in the upper troposphere. In addition, enhanced radiative heating due to the presence of more cirrus will increase the temperature near the tropopause, resulting in an increase in water vapor and upward motion, which leads to enhanced water-vapor flux into the stratosphere [125].

A third important effect of aerosols is their impact on stratospheric ozone through heterogeneous chemical reactions and through modifications of the large-scale circulation and temperature. Simulations show that variability in both water vapor and aerosols can induce stratospheric ozone variability [2,126,127]. As ozone-depleting substances continue to decrease as a result of the implementation of the Montreal Protocol, changes in halogen [3], water vapor and aerosols may become a much more important source of ozone variability in the future [126].

### Surface emission sources for the ASM-anticyclone chemistry

Surface emission sources and transport pathways are another important topic for the UTLS chemical processes. Simulation results show that the enhanced pollutants within the ASM anticyclone originate from the frequent deep-convection events, which transport the pollutants emitted from the surface to the upper troposphere (∼360 K). The fate of the air at the top of the convective regime, which is ascending diabatically in the anticyclonic monsoon circulation, is that they can be trapped there by the closed anticyclone circulation at the UTLS levels and remain for a long time [56,84,128]. The persistent deep-convection episodes during the Asian monsoon period are distributed over a wide area, including the South Asian subcontinent and the Bay of Bengal, the South China Sea and the Philippine Sea, the Tibetan Plateau and the southern slope of the Himalayas [94], most of which are far away from the ASM-anticyclone center. But pollutants (SO_2_ and NO_x_) from China have decreased since 2010 in response to the consequences of clean-air policy [60,129]. Research has been directed at identifying how pollutants are transported to the ASM anticyclone.

Bergman *et al.* [63] found a vertical conduit for the air in the summertime ABL to pass through the central troposphere and enter the ASM anticyclone. This conduit, centered over northeastern India, Nepal and the southern Tibetan Plateau, represents the most effective pathway for the ABL air to enter the ASM anticyclone. Obviously, the conduit lies between the ASM anticyclone and the strongest convective zone on the south side. As pointed out by Pan *et al.* [78], the fast upward transport related to deep convection in the Asian monsoon region is most effective from the surface to the upper troposphere, while subsequent transport to the lower stratosphere occurs mainly through the slow diabatic upwelling and the trapping effect of the ASM anticyclone [84,130]. The fast horizontal stirring and mixing effects caused by the intra-seasonal oscillation of the ASM anticyclone make these pollutants from the ABL diffuse and fully fill the whole anticyclone, but the upward movement is very weak during the ASM period [63,78].

The picture described above seems to depict a complete transport pathway from the ground to the ASM anticyclone; however, some important questions have not been clarified yet. First of all, where is the surface source for the air around the ASM anticyclone? According to the previous description, the concentration of pollutants within the anticyclone is higher than that in its periphery, so it is necessary to know how the sources in the ABL and the transport pathways differ for the air inside versus outside the anticyclone [94,97,131].

A second question concerns the distribution of surface pollutant sources for the monsoon air. It is well known that the dominant sources of anthropogenic pollutants are over the continents rather than over the oceans, but most of the studies in this field only discuss the geographical distribution of the pollutants [94,97,131], without considering the existence of pollutant emission. Although a few studies consider the pollutant emission, the available inventory is relatively too simple; for example, the spatial and temporal resolution needs to be improved or the types of emission sources need to be better defined [10,37,57,111]. In addition, the transport mechanisms are different over different source regions. Small-scale deep-convection events (Fig. 4) over the Tibetan Plateau are mainly caused by local convective instability [132,133], while the South Asian monsoon region is dominated by the Indian low-pressure system [50,51] and its associated with bulky deep convections (Fig. 4), and the convective transport over the ocean is mainly caused by tropical cyclones [64–66,71]. As for the contribution to the pollutants within the ASM anticyclone, preliminary simulations show that contribution from the Indian continental surface source is significantly larger than the contribution from the eastern Asian source, and that deep convective transport is particularly critical for upward transport of surface pollutants to the ASM anticyclone [7,37,57,78].

**Figure 5.**
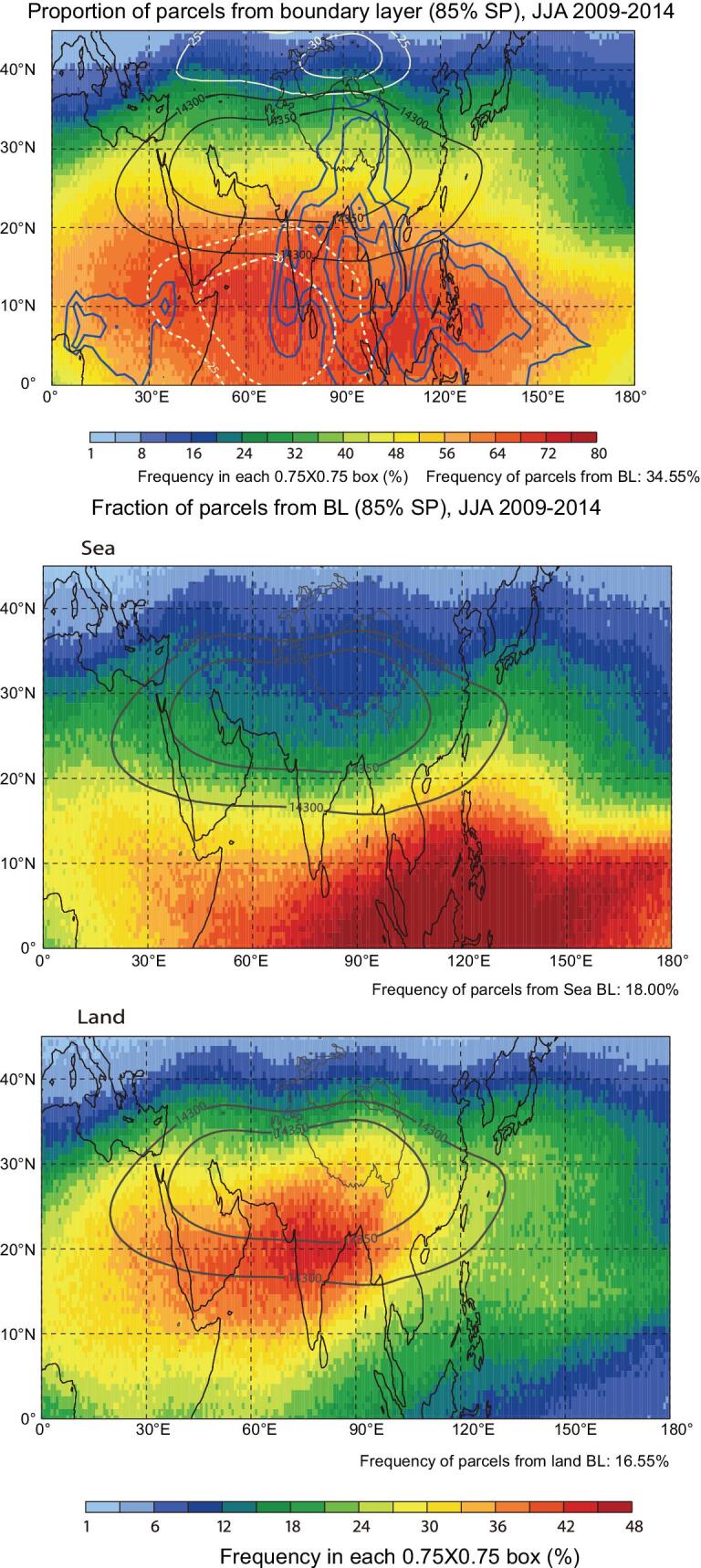
Percentage distribution (color scale) of air parcels at the 150-hPa level originating from all the ABL sources (upper), the oceanic sources (middle) and the continental sources (lower) within 30 days during summer, overlaid with JJA mean geopotential height (black contours; units: gpm), zonal wind (white contours; solid for westerly and dashed for easterly; units: m s^−1^) at 150 hPa and outgoing long-wave radiation (blue contours, for 220, 210, 200 and 190 W m^−2^). This figure is adapted from Fan *et al.* [131].

To address the above two questions, backward-trajectory calculations have been conducted to trace the ABL sources of the air at the 150 hPa level [131]. Although the ASM anticyclone is co-located with high concentrations of pollutants, trajectory simulations show that, within 30 days, the air at the 150-hPa level most frequently originates with ABL sources in the most intensive convection regions and their downwind areas (Fig. 5a), which are at the southern flank or periphery of the ASM anticyclone rather than at its center. The upper tropospheric air originates from two different ABL sources: one from the ocean with a dominant impact to the south of 20°N (Fig. 5b) and another from the continent with a dominant impact between 10°N and 30°N (Fig. 5c). The air from the continental ABL source makes the dominant contribution to the formation of the enhanced pollutant levels within the ASM anticyclone, while the clean air from the oceanic ABL sources dilutes the pollutants in the southeast part of the anticyclone [131]. This mechanism is somewhat different from the trapping effect as suggested above, and particularly the trapping effect is weak to the south of the anticyclone, which is evident from the occurrence of transport into the stratosphere [45,83,103] and intrusion into the anticyclone [64,71] from the south of the anticyclone.

## IMPACT ON THE GLOBAL STRATOSPHERE

The various surface pollutants entering the ASM anticyclone from lower levels can break through the anticyclone and diffuse to a wide area of the globe as mentioned before. Satellite observations show that the high concentration of water vapor within the ASM anticyclone during the boreal summer increases the water-vapor concentration in the lower stratosphere over the middle and high latitudes in the Northern Hemisphere in the following seasons (summer and autumn) [134,135]. Model simulations also show that the air within the ASM anticyclone can also enter the upward branch of the Brewer–Dobson circulation in the tropics [45,71,83,103].

In particular, a recent study shows that the emitted pollutants over Asia contribute to the stratospheric aerosols throughout the Northern Hemisphere [10]. The Community Aerosol and Radiation Model for Atmospheres sectional aerosol model coupled with the Community Earth System Model, after verifying the *in situ* measurement of the aerosol-size spectrum in the ATAL, is used to simulate the ATAL’s contribution to the global stratospheric aerosol layer and it is found that the ATAL aerosols diffuse to the whole lower stratosphere of the Northern Hemisphere. On average, the ATAL contribution accounts for about 15% of the Northern Hemisphere aerosols, which is comparable to all volcanic eruptions during the period 2000–15 [10]. A large amount of organic substances, sulfur and other substances (NH_3_) are emitted over Asia, and these precursors for aerosols are blown up into the tropopause layer by the ASM circulation. With the continuing growth of the Asian economy, the contribution of Asian enhanced pollutants to stratospheric aerosols will further increase [119].

## SUMMARY AND OUTLOOK

Satellite observations show that low values of total ozone are centered on the Tibetan Plateau every summer [12]. This summertime ozone valley is formed by two factors that contribute equally: the lower ozone concentration in the UTLS within the ASM anticyclone and the terrain-induced air-column shortage [32]. The first factor is caused by troposphere–stratosphere transport associated with the ASM circulation.

Satellite measurements as well as balloon soundings show enhanced tropospheric tracers and decreased stratospheric tracers within the ASM anticyclone [33]. These enhanced pollutants in the UTLS are from surface emissions, which are transported upward by frequent deep convection to the upper troposphere and then are trapped by the ASM anticyclone.

During the ASM period, deep-convection events include both local continental thunderstorms and remote strong tropical cyclones in the western part of the Pacific Ocean. Continental convection always carries a large amount of surface pollutants into upper levels, while deep convection from tropical cyclones brings ozone-poor air from the ocean surface to the UTLS and into the ASM anticyclone by horizontal advection [64,66]. Deep convection is crucial for very short-lived chemical species to enter the TTL because it provides quick transport from the surface sources [43].

The ASM anticyclone is an isolated circulation that can trap the inside air for a long time and prevent outside ozone-abundant and pollutant-poor air from mixing laterally into the anticyclone, and its intra-seasonal zonal propagation quickly mixes the inside pollutants horizontally. The ASM anticyclone plays a dominant role in the distribution of atmospheric constituents in the UTLS during the summer period [66,72,134,136–138].

Upper tropospheric air within the ASM anticyclone transports into the stratosphere through two main pathways. One pathway is over the southern part of the anticyclone via upward slow diabatic motions crossing the isentropic surfaces [45], which accounts for about two-thirds of the transport. Another pathway is over the northern and eastern parts of the anticyclone via isentropic transport by eastward and westward eddy shedding. The transport into the stratosphere via the ASM circulation is about two to three times more efficient than that from the North American summer monsoon circulation [103].

A significant phenomenon is the ATAL, which was discovered first by satellite measurements and confirmed later by balloon soundings. A robust enhancement in aerosol concentration extends up to 2 km above the tropopause, with a particle surface area density peak at ∼0.18-μm diameter. The formation mechanism for the ATAL is unsettled due to the limited observations of the chemical composition of the aerosols. Preliminary *in situ* measurements show the existence of gas-phase ammonia and solid ammonia nitrate within the ATAL [121]—an unexpected and surprising finding.

The atmospheric composition within the ASM anticyclone has impacts on regional climate through microphysical and radiative processes, and affects the budget of the stratospheric constituents (such as aerosol, water vapor and ozone) outside the anticyclone.

Although some advances have been achieved in the study of Asian surface pollutant transport to the north-hemisphere stratosphere over the Tibetan Plateau, there are still some challenges for further understanding.

The first challenge comes from the understanding of deep convection. The geographical distribution, frequency and diurnal variation of deep convection over the ASM region are important for investigating pollutant transport from various surface sources and moisture transport into the UTLS, particularly for penetrating convection events. Deep convective clouds transport air masses from the surface to the upper troposphere within several hours, which is important for short-lived chemical species (e.g. NH_3_) to enter the TTL [43,44,121]. Deep convective clouds play a key role in water-vapor variation, which will impact the microphysical process in the TTL within the ASM [48,65]. Therefore, more measurements from satellite-borne cloud radars and ground-based cloud/weather radars with higher spatial and temporal resolution, and more advanced approaches to derive the properties of deep convection, are needed.

The second challenge comes from the understanding of microchemistry and microphysics processes related to the convection. Measurements show the existence of gas-phase ammonia and solid ammonium nitrate in the UTLS over the ASM region [121], which is expected to be washed out by the convection and therefore not transported to the upper troposphere. Therefore, microphysical and microchemical processes in clouds during deep convection should be investigated further, particularly for the transport of soluble gases and water vapor. Deep convection over some heavily polluted regions, such as Sichuan Basin and northernmost India, should receive more attention and further study.

The third challenge comes from the understanding of the ATAL. The composition of the aerosol within the ATAL is still a mystery. Although some preliminary *in situ* and remote measurements of the aerosol composition have been made [121], all of them are over the southern part of the ASM anticyclone, where the pollutants are freshly transported by fast deep convection from the surface layer and therefore are possibly different from aerosols in the northern part of the anticyclone, which has more aged air. The formation mechanism of the ATAL still remains unsolved, along with other issues such as the nucleation mechanism, transport mechanism and emission inventory for various constituents. The impacts of the ATAL on cirrus formation, chemistry and radiative forcing are also topics for future investigation.

The last challenge comes from the *in situ* measurements. Some *in situ* measurements have been conducted, such as the early balloon-borne ozonesonde [16–18] and aerosol counter [139] observations in the mid-1990s in Xining and Lhasa over the Tibetan Plateau; balloon soundings of water vapor, ozone, particle at several stations over the Tibetan Plateau [34,140,141], India and Saudi Arabia [107] and southern slopes of the Himalayas [141]; lidar measurements over the Tibetan Plateau [110,142]; CARIBIC (civil aircraft for the regular investigation of the atmosphere based on an instrument container) campaign [143]; and high-altitude aircraft measurement flights over South Asia by the StratoClim project (http://stratoclim.org). These measurements have provided many interesting results, such as the ammonium-nitrate aerosol layer being formed via transporting ammonia from surface sources into the upper troposphere by convection [121]. However, due to various limiting conditions, there are still some critical problems unresolved, such as the composition and size distribution of aerosol within the ATAL (particularly for aged aerosols), microphysical parameters of cirrus in the upper troposphere, concentrations for some very short-lived species important for ozone chemistry in the UTLS and temperature perturbations critical for cirrus formation in the upper troposphere.
